# Managing Marine Plastic Pollution: Policy Initiatives to Address Wayward Waste

**DOI:** 10.1289/ehp.123-A90

**Published:** 2015-04-01

**Authors:** John H. Tibbetts

**Affiliations:** John H. Tibbetts, based in Charleston, SC, is the former editor of *Coastal Heritage*, the magazine of the South Carolina Sea Grant Consortium.

A few times a year, volunteers fan out along the causeway that links the South Carolina mainland with the seashore community of Folly Beach to clean up plastic bottles, straws, bags, and other debris from along the road and the salt marsh. Some of this debris has come from cities miles away. On windy days, litter is often blown off city streets into waterways. During rainstorms, debris floats into drains that empty into rivers. Other trash probably came from places closer to home. “I see bags and other plastic flying off the beds of pickup trucks going down the causeway,” says Marty Morganello, who organizes the cleanups for the Charleston-area chapter of the nonprofit Surfrider Foundation. “I see them coming out the open windows of cars and out the backs of garbage trucks and even recycling trucks. This material is lightweight, and if you don’t secure it, it will fly away.”

**Figure:**
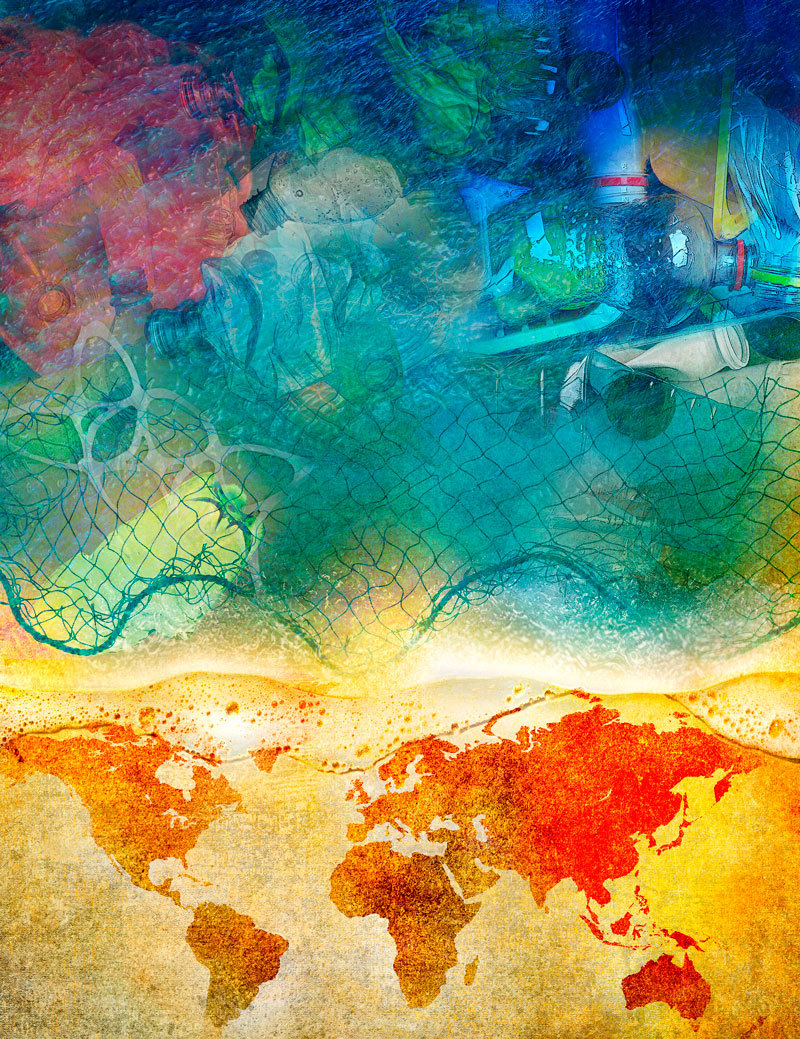
By one estimate, the volume of plastic debris going into the world’s oceans could more than double by 2025, assuming current trends in coastal development and plastics use. Some countries have begun identifying ways to improve management of plastic waste, creating solutions that make sense both for business and for the sea. © Roy Scott

Beach cleanups yield enormous amounts of trash, with plastic items a major constituent.^1^ Although the human health impacts of this marine plastic pollution remain poorly characterized, it is widely seen as an emerging problem that deserves much more research attention.^2^ Likewise, there is a growing urgency among industry, government, nongovernmental organizations, and environmental groups to develop tools and policies to track, capture, and recycle plastic waste before it reaches the ocean.

## Sources of Marine Plastic Pollution

Lost and discarded nets and lines from fishing vessels are important contributors to marine debris, especially in heavily fished areas. These vessels also lose plastic floats, traps, pots, and other gear. Other sea-based sources of plastic pollution include oil and gas platforms, aquaculture facilities, and cargo ships that lose containers to the sea.^3^

Plastic debris from land comes primarily from two sources: first, ordinary litter; and, second, material disposed in open dumps or landfills that blows or washes away, entering the ocean from inland waterways, wastewater outflows, and the wind.^4^ Major waterways can transport a great deal of plastic waste. One study estimated that the Danube River, for example, transports 4.2 metric tons of plastic into the Black Sea each day.^5^

Lightweight plastic items tend to float in water and can be carried by currents great distances. By one report, plastic cargo lost from ships has been found more than 10,000 kilometers from where it was lost.^6^ Likewise, currents can carry floating fishing nets hundreds of miles from where they were last used, according to Nancy Wallace, director of the National Oceanic and Atmospheric Administration’s (NOAA) Marine Debris Program. The Northwestern Hawaiian Islands do not have significant fishing nearby—they lie within the largest marine wildlife reserve in the world—but in 2014 NOAA-supported collection efforts there rounded up about 52 metric tons of lost nets and other plastic debris.^7^

A working group of researchers recently estimated that just 20 countries, out of a total of 192 with coastlines, are responsible for 83% of the plastic debris put into the world’s oceans. Lead author Jenna R. Jambeck, an environmental engineer at the University of Georgia, and her colleagues estimated that, all together, these 192 countries produce some 275 million metric tons of plastic waste each year. Of that volume, about 4.8–12.7 million metric tons of mismanaged plastic waste is thought to have entered the ocean in 2010.^4^

“That is the same as five five-gallon bags filled with mixed plastic on every foot of coastline around the world,” says Jambeck. Without improvements to waste management infrastructure, and assuming a business-as-usual projection of increasing coastal populations, economic growth, and use of plastics, the authors predict this volume of plastic debris could more than double by 2025.^4^

## The Impact of Coastal Countries

**Table:** Table of top 20 countries in terms of mismanaged plastic waste

Rank	Country	Percentage of waste that is mismanaged	Quantity of mismanaged plastic waste (MMT/year)	Percentage of global mismanaged plastic waste	Quantity of plastic marine debris (MMT/year)
1	China	76	8.82	27.7	1.32–3.53
2	Indonesia	83	3.22	10.1	0.48–1.29
3	Philippines	83	1.88	5.9	0.28–0.75
4	Vietnam	88	1.83	5.8	0.28–0.73
5	Sri Lanka	84	1.59	5.0	0.24–0.64
6	Thailand	75	1.03	3.2	0.15–0.41
7	Egypt	69	0.97	3.0	0.15–0.39
8	Malaysia	57	0.94	2.9	0.14–0.37
9	Nigeria	83	0.85	2.7	0.13–0.34
10	Bangladesh	89	0.79	2.5	0.12–0.31
11	South Africa	56	0.63	2.0	0.09–0.25
12	India	87	0.60	1.9	0.09–0.24
13	Algeria	60	0.52	1.6	0.08–0.21
14	Turkey	18	0.49	1.5	0.07–0.19
15	Pakistan	88	0.48	1.5	0.07–0.19
16	Brazil	11	0.47	1.5	0.07–0.19
17	Burma	89	0.46	1.4	0.07–0.18
18	Morocco	68	0.31	1.0	0.05–0.12
19	North Korea	90	0.30	1.0	0.05–0.12
20	United States	2	0.28	0.9	0.04–0.11
MMT = million metric tons Adapted from Jambeck et al. (2015)^4^					

The United States makes a significant contribution to marine plastic pollution, but it’s only twentieth on the list of coastal nations that produce the most plastic waste from land. The top spots are filled by a number of rapidly developing countries with expanding populations near coastlines and poor systems of waste management, including China, Indonesia, and the Philippines.^4^

One of the major drivers of this trend in developing countries is the very rapid growth of “megacities,” defined as urban areas with populations exceeding 10 million. More than 70% of megacity growth is said to occur outside the formal planning process, and nearly a third of the urban population in developing countries lives in slums or informal settlements that lack city services, including solid-waste disposal.^8^

According to Jambeck and colleagues, a nation’s population density within 50 kilometers of the coast is the primary determinant of its land-based contribution to marine pollution.^4^ For instance, about 74% of Indonesia’s population and 83% of the Philippines’ population live in coastal regions.^9^ The second determinant is how much waste overall a coastal nation produces on a per-capita basis. At 2.58 kilograms per person per day, the United States produces far greater volumes of waste per capita than any other nation on the top-20 list except Sri Lanka, and more than twice as much as China.^4^

The third determinant is how much of a country’s waste, including plastic material, is mismanaged. The United States does well on that score. “U.S. mismanaged waste is only due to litter,” says Jambeck. “We have a waste-management infrastructure that allows everyone an opportunity to throw something away properly.” China’s coastal population is about 2.5 times larger than that of the United States but is estimated to produce more than 30 times more mismanaged plastic waste.^4^

The geographies of countries play an important part in their contribution to marine debris. Among the top 20 ocean polluters are Sri Lanka, an island nation; archipelago countries, such as the Philippines and Indonesia; and countries with long coastlines, such as China and Vietnam.^4^

“This study [by Jambeck et al.] provides a first cut at how you could focus efforts in places around the world and then build some strategies to stem that flow of plastics,” says George H. Leonard, chief scientist of Ocean Conservancy, an advocacy organization based in Washington, DC. “Marine debris is a global problem, but this study shows that you can work on a smaller suite of geographies [and] that you could solve a big part of the problem at the global level.” The key, he says, is to improve waste management in a relatively small number of countries.

## Extended Producer Responsibility

Some European nations have developed a model that other countries and regions could emulate to better manage their plastic waste and reduce marine pollution. It is based on the principle of extended producer responsibility (EPR), which was first formally outlined in an internal Swedish government report in 1990.^10^ The idea behind EPR is to shift financial responsibility for end-of-life disposal to product manufacturers, thereby providing an incentive for improved product design, reuse, and recycling.^11^

In an EPR scheme, brand owners must pay the costs of tracking, managing, and recycling or disposing of packaging after their products have been used.^11^ EPR is usually implemented through take-back legislation that requires manufacturers to recover their packaging after product consumption. Some producers pay a fee to organizations that collect and recycle the packaging. Container-deposit systems that some U.S. states have for soda bottles are one example of an EPR initiative.^12^

Many European nations have not only passed EPR laws to increase reuse and recycling of plastics but also are diverting plastics to power plants for use as fuel for heat and electricity (a process called waste-to-energy, or WTE).^13^ In Europe, an estimated 25.2 million metric tons of post-consumer plastic was discarded in 2012, according to the manufacturers association PlasticsEurope.^14^ Of that amount, 26% was recycled, 36% was recovered for fuel, and 38% went to landfills.^14^ In 2012 the United States produced approximately 29 million metric tons of post-consumer plastic waste but recycled only 9% of it and used perhaps 16% for fuel.^15^

Nine European nations have banned landfills in part because available land is scarce in their densely populated areas. One result of this legislative decision is that 90–100% of plastics are recycled or used for energy production in these countries. But several other countries still landfill more than 60% of their waste, and some of these, especially in Eastern Europe, still depend totally on landfills. PlasticsEurope is calling for zero plastic waste going to European landfills by 2020.^14^^,^^16^

“Europeans have developed pretty robust recycling and energy recovery systems to manage their plastic waste,” says Steve Russell, vice president of the plastics division of the American Chemistry Council. “A primary driver for those systems has been the desire to capture energy and to remain as energy-independent as they can [while also dealing] with the landfill bans.” The United States, by contrast, has more acreage to build landfills and much lower prices for conventional energy sources, Russell says. “Whatever systems we design in the U.S.,” he says, “need to reflect the local conditions we have here.”

**Fate of Plastics in the Ocean**

Plastics in the ocean degrade into smaller pieces from the effects of sunlight, oxidation, and the abrasion of waves and currents, becoming smaller and smaller often to the point they are no longer visible to the naked eye.^22^ Marine organisms from zooplankton to fish consume these so-called microplastics, mistaking them for food.^23^,^24^,^25^

The consumption of plastic by marine organisms adds persistent, bioaccumulative, and toxic substances to the aquatic food chain.^26^,^27^ However, it is not clear what the net effect of plastics may be in either transferring persistant pollutants or reducing their bioavailability. Participants at a recent workshop convened by the U.S. Environmental Protection Agency concluded that the state of the science does not currently allow an assessment of possible human health risks from the ingestion of seafood contaminated with microplastics.^28^

## The Economics of Plastic Waste

For years, China was the primary buyer for low-quality “mixed bales” of plastic scrap—often contaminated with food, dirt, and nonrecyclable materials—that do not have a commercial market in the United States. China converts much of this scrap into feedstock, or resin, for its expanding manufacturing sector. But some portion of the scrap can’t be recycled and ends up in China’s landfills.^17^

In 2013 China enacted its so-called Green Fence operation to improve quality controls and stem the import of low-quality plastic scrap that would have to be landfilled. While exports of U.S. plastic scrap to China fell 18% from 2012 to 2013,^18^ the U.S. plastics recycling industry responded to Operation Green Fence by updating its facilities to produce a “cleaner, more consistent bale,” says Keith Christman, managing director of plastics markets for the American Chemistry Council. “The quality of recycled plastic has gone up, so the market has remained strong and recovered,” Christman says. “As the markets have grown and the ability to separate materials has grown, so those changes have come together to provide more value, and more market, for that material.”

Despite China’s restrictions, U.S. plastics recycling continued to grow in 2013, with plastic bottle recycling up by 4.3% over 2012,^19^ and polyethylene film recycling up by 11%.^20^ Recycling of non-bottle rigid materials (e.g., yogurt tubs, clamshell containers) declined by just under 1% in 2013 but overall has tripled since 2007 to more than 1 billion pounds per year as more communities have added non-bottle rigid containers to their collection programs.^20^

Plastic polymers are almost completely derived from petrochemicals, depending on which feedstock is most cost effective at the point where the feedstock is produced. (According to Christman, more than 70% of U.S. plastics are made from domestic natural gas.) Plastic recycling increases when it’s cheaper to create resin from recycled waste that it is to create it directly from fossil fuels, says Jim Glauser, a specialty chemicals expert and associate director at IHS, a U.S.-based information business. To improve recycling rates, “you need to improve the collecting, sorting, and processing of plastic waste to lower the cost and improve the quality of resin from recyclables,” Glauser says.

## Seeking Solutions

Over the coming decades, the volume of plastic waste moving from the land into the sea is expected to increase if the many coastal economies and populations around the world continue to expand without taking steps to manage their municipal solid waste.

Marine plastic pollution will remain a difficult problem to solve because it represents a “fundamental market failure” on a worldwide scale, says Leonard of Ocean Conservancy. He explains, “The production of plastic is ramping up,^14^ but society isn’t able to keep up with that waste.”

NOAA’s Wallace believes gear markings or a global system for reporting lost gear would be helpful in managing sea-based plastic pollution. “Today, we can’t trace any of that gear back [to its source],” she says. “It would be good to know which countries many of these nets are coming from, so we could find practices that would stop it from happening in the first place.”

In 2012 Ocean Conservancy mobilized a new effort called the Trash Free Seas Alliance®, which includes chemical and plastics companies, producers of plastic consumer items, economists, environmental scientists, and conservation groups. The alliance is using the cross-sector expertise of its members to develop innovative, sustainable strategies to eliminate ocean waste.^21^

“We need a better understanding of the economic restraints to solve the plastic debris problem,” Leonard says. “We are looking at business practices that could allow greater recapture, recovery, reuse. Then we can identify and craft a suite of locally relevant solutions that make sense for business and for the ocean.”
